# Survey of German pet owners quantifying endoparasitic infection risk and implications for deworming recommendations

**DOI:** 10.1186/s13071-019-3410-2

**Published:** 2019-05-03

**Authors:** Christina Strube, Ann Neubert, Andrea Springer, Georg von Samson-Himmelstjerna

**Affiliations:** 1 Institute for Parasitology, Centre for Infection Medicine, University of Veterinary Medicine Hannover, Buenteweg 17, 30559 Hannover, Germany; 2 Elanco Deutschland GmbH, Werner-Reimers-Strasse 2-4, 61352 Bad Homburg, Germany; 3 Present Address: Fridolinweg 5, 12683 Berlin, Germany; 4 Institute for Parasitology and Tropical Veterinary Medicine, Freie Universität Berlin, Robert-von-Ostertag-Strasse 7-13, 14163 Berlin, Germany; 5 ESCCAP Deutschland e.V., c/o Vetproduction, Domstraße 28, 50668 Koeln, Germany

**Keywords:** ESCCAP, Risk assessment, Zoonosis, *Toxocara*, *Echinococcus*, Parasite control, Dogs, Cats

## Abstract

**Background:**

Dogs and cats can transmit zoonotic helminths to humans, e.g. *Toxocara* spp. and *Echinococcus multilocularis*. Strategic deworming may help minimize this risk. Studies in several European countries have shown that pets are dewormed less frequently against roundworms and tapeworms than recommended by the European Scientific Counsel Companion Animal Parasites (ESCCAP). The objective of this study was to identify percentages of dogs and cats falling into the different risk categories defined by the German ESCCAP guidelines and to evaluate whether deworming frequency and parasite monitoring in Germany follows these guidelines.

**Results:**

According to questionnaire results from 500 dog and 500 cat owners, deworming of dogs in Germany averages 2.07 times/year while for cats this average is 1.72 times/year. In contrast, evaluation of risk factors placed only 2% (10/500) of dogs in ESCCAP category A with a recommended deworming/examination frequency of 1–2 times per year, while 4.8% (24/500) were placed in category B (4 treatments/examinations per year recommended), 30.8% (154/500) in category C (12 treatments/examinations per year against tapeworms and 4 treatments/examinations per year against roundworms recommended) and 62.4% (312/500) in category D (12 treatments/examinations per year recommended). All cats were placed either in risk group A [52.8% (264/500)] or D [47.2% (236/500)]. Generalized linear models indicated that risk group D cats were treated significantly more often against helminths than risk group A cats. There were no significant differences in deworming frequency between risk groups in dogs. The most important factor influencing deworming frequency was the frequency of veterinary visits. Dogs and cats were treated significantly more often if owners visited their veterinarian more than once yearly.

**Conclusions:**

The percentage distribution of risk groups considerably varied between dogs and cats. Nevertheless, 62% of dogs and 47% of cats were assigned to category D for which monthly treatments/examinations are recommended by the ESCCAP guidelines. Veterinarians play a key role in instructing pet owners with regard to helminthoses and their prevention, and should take the time for adequate risk assessments. The reported low deworming frequencies despite the high potential parasite infection risk suggests that pet owner advice through veterinarians needs to be improved.

## Background

According to a risk assessment performed by the German Public Health Institute (Robert Koch Institute), the close relationship of people to their companion animals provides more benefits with regard to socialisation, mental and physical health, than risks [1]. Nevertheless, there is a possibility for infection with and transmission of zoonotic companion animal parasites such as *Toxocara* spp. and *Echinococcus multilocularis* within Germany. Human behaviour such as hand hygiene, prevention of environmental contamination (e.g. pets being denied access to children’s playgrounds, cleaning up of dog faeces from soil), education of the public and use of strategic anthelmintic treatment may help to minimize the risk for zoonotic diseases [2].

However, several studies have revealed that European pet owners are not aware of the public health risks posed by helminths and the possibility of disease transmission from their dogs and cats to themselves [3–7]. This could lead to the conclusion that the vast majority of pet owners do not request the recommended strategic worm diagnostics nor perform the recommended anthelmintic treatments. In studies conducted in the Netherlands, only 24.5% of cats [6] and only 16% of dogs were dewormed four times a year [3]. To the knowledge of the authors, no similar data have been published for Germany so far. Therefore, the objective of this study was to evaluate whether current deworming behaviour in Germany follows the accepted guidelines set forth by the German chapter of the European Scientific Counsel Companion Animal Parasites (ESCCAP) for the control of tapeworms and roundworms.

Human alveolar echinococcosis, caused by the larval stage of *E. multilocularis*, is considered as the most dangerous autochthonous parasitic zoonosis in Germany. According to a recent meta-analysis, Germany is among the “high prevalence” countries with a pooled prevalence > 10% of *E. multilocularis* infections in red foxes, the main definitive host [8]. The parasite is endemic in Germany with highest prevalences in southern federal states [9], but also shows significant prevalences in the northern part of Germany, e.g. in Brandenburg [10], Lower Saxony [11] and Schleswig Holstein [12]. Taking into account that a distinct increase in fox populations, particularly in urban areas, has been observed in Europe and that the public intensively uses these areas, foxes could play an important role for transmission of human alveolar echinococcosis and may represent a reservoir from which spillover to companion animals can occur [13]. In humans, there is a heterogeneous case distribution of alveolar echinococcosis throughout Germany, with most cases reported from the federal states Baden-Wuerttemberg and Bavaria [14]. For example, 18 of 26 cases were reported from these states in 2016, although it is important to consider that the place of residence of the patient does not necessarily reflect the place of infection [15]. The pooled prevalence in Germany is 0.3% in dogs and 0.6% in cats [8]. Due to different methodologies used for detection, the data for foxes and pets are not entirely comparable [sedimentation and counting technique (SCT), intestinal scraping technique (IST), copro-antigen ELISA or PCR in foxes *vs* mainly flotation as a less sensitive method and confirming PCR in pets]. Nevertheless, dogs should be recognized as relevant hosts that can introduce *E. multilocularis* into non-endemic areas by travelling from endemic to non-endemic regions with their owners [8]. Indeed, there is concern that the risk for humans to acquire alveolar echinococcosis may rise due to the suspected geographical spread of the parasite [16]. Dogs might also play an important role in zoonotic transmission of alveolar echinococcosis due to their close association with humans [8]. Based on data from an experimental infection study the reproductive potential of *E. multilocularis* in cats is low, thus their relevance is also considered to be low [17]. However, there is a hint that the role of cats in the *E. multilocularis* life-cycle and in transmission of alveolar echinococcosis may currently be underestimated [18]. Amongst other risk factors like vocational factors (e.g. being a farmer or handling foxes), human habits (e.g. chewing grass) and socio-cultural factors (e.g. belonging to a certain ethnic group, having a low income), “dog ownership”, “play with dogs” and “cat ownership” are important potential risk factors for humans acquiring alveolar echinococcosis [19].

Increased travel activity of pet owners with their pets brings the possibility of transmission of distant parasites to the park next door. A recent study on urban dog parks in Lisbon, Portugal, highlights the potential of these parks as a source of transmission for canine parasites, including some with zoonotic potential [5]. For example, *Toxocara* spp., the roundworms of dogs and cats, may pose a risk to humans. Upon ingestion of embryonated *Toxocara* eggs present in the environment or larvae contained in undercooked meat of paratenic hosts, the clinical syndromes of *larva migrans visceralis*, ocular toxocarosis, neurotoxocarosis or covert toxocarosis may develop in humans and possibly lead to long-term health consequences [20]. Eggs of *Toxocara* spp. are the most frequently found helminth eggs in diagnostic faecal samples of dogs and cats in Germany [21] and have recently been found as contamination in up to 40% of children’s playgrounds in the northern German city of Hanover [22].

In addition, not only zoonotic parasites are of concern, but also parasites that cause severe diseases in dogs and cats. The pets’ risk of infection with specific helminths in formerly non-endemic regions has grown due to environmental and human behavioural changes, e.g. movement of dogs [23–25]. Indeed, there is an indication for a significant increase of *Angiostrongylus vasorum* and *Crenosoma vulpis* prevalences from 2003 to 2015 and a potential expansion of *A. vasorum* endemic areas to the northeastern part of Germany [23]. The diagnosed prevalence of *A. vasorum*-infected dogs varied between 0.01 and 8.7% with the highest prevalence in Baden Wuerttemberg, Rhineland-Palatinate, Saarland, North Rhine-Westphalia, Berlin and Brandenburg [23]. Furthermore, the first autochthonous case of *Dirofilaria repens* in Germany was described in the region of Karlsruhe in 2006 [26] and a possible endemisation of this parasite in the Havelland region is discussed [25].

Studies identifying risk factors for acquiring parasite infection with roundworms and tapeworms are summarized in the ESCCAP guidelines and their German adaptation [27, 28]. These guidelines aim to protect both the health of the pet as well as the health of the public by reducing the risk of zoonotic parasite transmission [4]. ESCCAP guidelines recommend a worm control regime designed specifically for each pet based upon an individual assessment of risk factors [28]. A helpful tool for veterinarians to estimate the individual risk and recommend faecal analysis or deworming frequency is a flow chart developed by ESCCAP that takes these risk factors into consideration. The following risk factors are considered in the flow chart: pet goes outdoors without supervision, contact with other animals not from the same household, coprophagia or feeding on carcasses, hunting or feeding on prey. Additional diagnostic or treatment recommendations are given for puppies/kittens, pregnant and lactating bitches/queens, exhibitions, sports competitions and kenneling, professional use in therapy or as a police dog, close contact with children or immunosuppressed persons, travelling and feeding on raw meat.

Little information is available on the percentage of German dogs and cats that fall into each parasitic risk category as defined by ESCCAP. Therefore, the aim of this study was to identify how many dogs and cats fall into each risk category and investigate whether there are significant differences in deworming behaviour among these risk groups. A study that outlines the European situation in an overview has been recently published as part of this collection [29]. The present study focuses on the current situation with relevant parasites in Germany. In contrast to the data presented by McNamara et al. [29], the present study defines risk groups based on the current German adaptation of the ESCCAP guidelines, which differ from the European guidelines, as protection against *A. vasorum* is not (yet) included in the risk assessment. Furthermore, apart from ESCCAP risk groups, several other factors that might influence deworming behaviour in Germany are examined.

## Methods

### Study design

From 3rd July 2017 to the 14th July 2017 an online survey was conducted among cat and dog owners in five European countries. Details on the target group, inclusion and exclusion criteria can be found in McNamara et al. [29]. Here we present the data that were collected from Germany.

A total of 18,020 German pet owners were contacted by email in order to achieve a target sample of 500 dog owners and 500 cat owners.

The place the participants lived in was defined as rural area (area completely away from a major city, such as a village or countryside), town (town centre or close to a town/small city), suburban - metropolitan area (within a few miles of a city centre/urban area/large city) or city - metropolitan area (city centre/urban area/large city).

In the survey, the question concerning deworming frequency was deliberately placed first to ensure that the subsequent questions did not affect the answer of the participant. Questions about the pet’s lifestyle (e.g. pet’s age, outdoor access, living with children/elderly, see [29]) followed and information was matched with a risk assessment questionnaire that was designed based on the German ESCCAP guidelines. In contrast to the risk assessment used by McNamara et al. [29], the factor “living with children” was not taken into account when assigning risk groups, because “children” was defined as “aged 17 and under”. ESCCAP recommendations relate to “small children” but no detailed data on the age of the children were available. Additionally, the risk factor “eating grass” was not considered for risk group assignment in dogs nor in cats, because it is not part of the current German ESCCAP risk assessment. In Germany, “garden-only” access for cats is uncommon. Furthermore, if an outdoor cat has contact with other cats not from the same household, this is usually unknown to the pet owner. Therefore, these questions were not asked for cats. The information on pet lifestyle and exposure risks placed the pet into one of four distinct ESCCAP risk groups (A, B, C or D), for which different worm diagnosis or deworming frequencies are recommended (Table 1).

**Table 1:** Risk group definitions according to German ESCCAP guidelines for animals, without consideration of special risk factors (e.g. puppies, kittens, animals used for exhibitions)

Risk group	Description	ESCCAP recommended faecal examination or deworming frequency against roundworms and tapeworms
A	Lives indoors only or goes outdoors but has no direct contact with dogs and cats of other households and does not eat prey animals/raw meat, carrion or faeces	1–2 times per year
B	Goes outdoors under supervision and has direct contact with dogs and cats of other households; but does not eat prey animals/raw meat, carrion or faeces	4 times per year
C	Goes outdoors under supervision and has direct contact with dogs and cats of other households and eats prey animals/raw meat, but does not eat carrion or faeces	4 times per year against roundworms, 12 times per year against tapeworms
D	Goes outdoors without supervision or under supervision, but has direct contact with dogs and cats of other households and eats carrion or faeces	12 times per year

In addition, questions were asked with regard to the owner’s attitude towards their pet as well with regard to their sources of information on deworming. Finally, pet owners were presented with a list of anthelmintic formulations for dogs and cats currently licensed in Europe and asked which of these they had used during the past 12 months.

### Statistical analyses

The distribution of ESCCAP risk groups among animals resident in cities, suburbs, towns and rural areas was compared using pairwise Fisher’s exact tests, followed by Bonferroni correction of *P*-values.

Annual deworming frequency of dogs and cats was compared using the Wilcoxon-Mann-Whitney U-test. For each species, factors influencing annual deworming frequency were assessed in general linear models (GLMs) with Poisson error structure and log-link function using the package lmerTest [30] in R v.3.3.1 [31]. The following factors were included: owner gender, owner age, the owner’s attitude towards their pet (affectionate; devoted; dispassionate; sceptical), annual vet visits (once a year only; more than once a year), the animal’s risk group according to the German ESCCAP guidelines, neighbourhood (rural; town; suburb; city) as well as whether or not the owner sought information on deworming (from veterinary personnel, non-veterinarians and books/ magazines). Allocation to four groups of pet owners’ attitude was performed according to the evaluation of pet owners’ degree of agreement on six statements about companionship. Full models were compared to null models containing only an intercept term in a likelihood ratio test (R-function “anova”, test = ”chisq”). Model assumptions were validated graphically by inspecting histograms and qq-plots of residuals as well as residuals *vs* fitted values and residuals *vs* predictor variables. Multiple comparisons between the levels of “neighbourhood” and the levels of German ESCCAP risk group (dogs only) using Tukey contrasts with single-step *P*-value adjustment were conducted using the “glht” function of the R package *multcomp* [32].

Initially, questionnaire results on the pet owners’ attitude towards anthelmintics were included in the GLMs. However, no statistically significant association with deworming frequency was found. Thus, they were subsequently excluded, which improved overall model fit.

## Results

### Dogs

Among the 500 completed dog questionnaires, the most frequently reported risk factors for dogs were contact with children/elderly (91%), contact with other dogs, snails or prey (89%) and going off-lead (76%). Only 14 dogs were under 6 months-old. Details are provided in Table 2.

**Table 2:** Results of dog and cat questionnaires including percentages as shown in McNamara et al. [29]

	Dog dataset (*N* = 500)	Cat dataset (*N* = 500)
Owner gender	318 female, 182 male	311 female, 189 male
Mean owner age ± SD (range) in years	44.5 ± 13.62 (18–81)	44.5 ± 13.96 (18–78)
Animal > six months of age, *n/N* (%)	486/500 (97.2)	493/500 (98.6)
Contact with children/elderly, *n/N* (%)	455/500 (91.0)	336/500 (67.2)
Kept only indoors, *n/N* (%)	na	249/500 (49.8)
Goes outdoors, but garden only, *n/N* (%)	110/500 (22.0)	na
Goes off-lead (those that go outside the garden), *n/N* (%)	296/390 (75.9)	na
Hunts (those that go outdoors), *n/N* (%)	na	222/251 (88.4)
Catches prey (those that go outdoors), *n/N* (%)	95/500 (19.0)	214/251 (85.3)
Contact with dogs of other households, snails or prey, *n/N* (%)	446/500 (89.2)	na
Eats slugs, snails, grass or digs in garden, *n/N* (%)	334/500 (66.8)	na
Eats raw meat (those that do not go outside unsupervised or catch prey), *n/N* (%)	158/405 (39.0)	90/286 (31.5)
Mean no. of annual dewormings ± SD (range)	2.1 ± 1.42 (0–12)	1.7 ± 1.33 (0–12)

*Abbreviations*: n, number of positive answers; N, number of people questioned, na, not applicable (dog only or cat only questions, respectively)

**Fig. 1:**
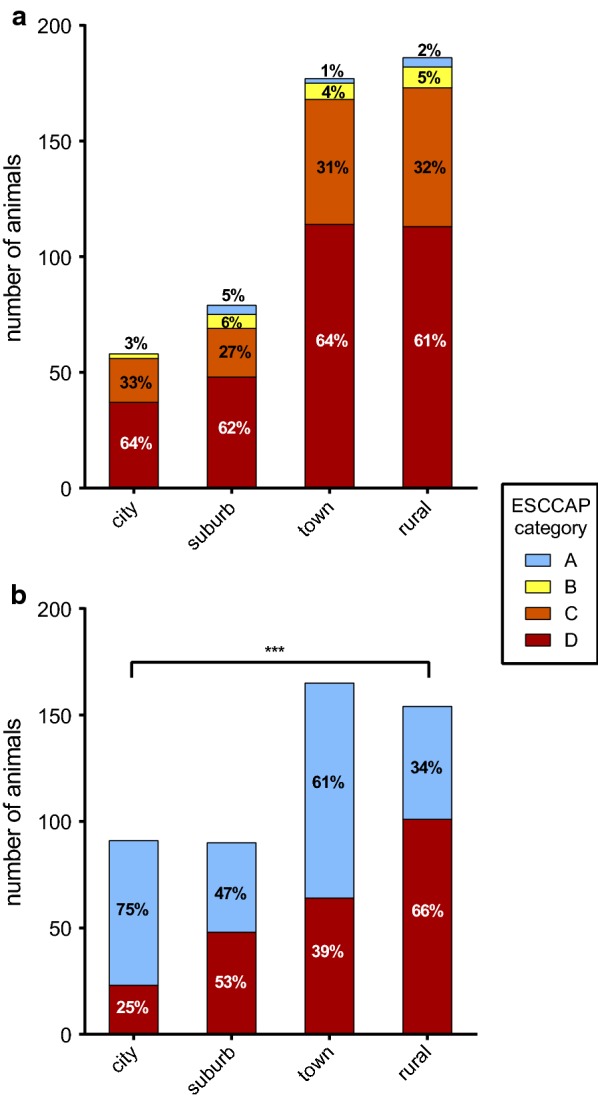
Distribution of ESCCAP risk groups in different neighbourhood categories in **a** dogs and **b** cats. *** *P* < 0.001

**Fig. 2:**
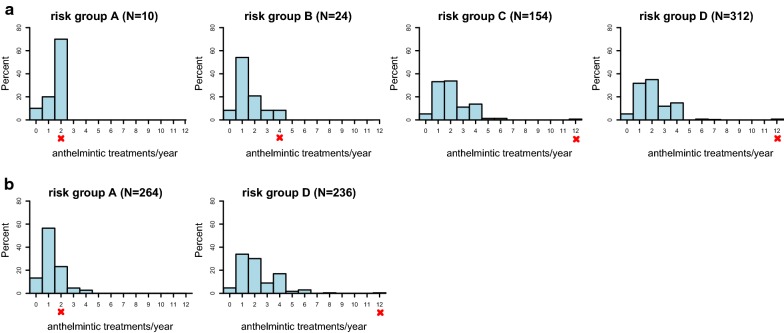
Distribution of annual deworming frequency according to ESCCAP risk group in **a** dogs and **b** cats. The red cross indicates the frequency of treatment against tapeworms for each risk group as recommended by ESCCAP

According to German ESCCAP guidelines, only 2.0% (10/500) of dogs were placed in category A with a recommended examination/ deworming frequency of 1–2 times per year, while 4.8% (24/500) were placed in category B (4 examinations/ treatments per year recommended), 30.8% (154/500) in category C (recommendation of 12 examinations/ treatments per year concerning tapeworms and 4 treatments per year against roundworms) and 62.4% (312/500) in category D (12 examinations/ treatments per year recommended). No significant difference in the distribution of risk groups for dogs kept in cities, suburban areas, towns or rural areas was found (Fig. 1a). The average number of dewormings per year reported in this survey in dogs was 2.07 ± 1.42 (mean ± standard deviation, SD). The distribution of deworming frequency per ESCCAP risk group is depicted in Fig. 2a. In total, 97.6% (488/500) of dogs were treated less often than recommended based on their risk group assignment. Ten dog owners (2%) reported that they treated their dog more than 4 times per year, while 25% (125/500) treated their dog 3–4 times per year. Most dog owners (84.2%, 421/500) indicated that they believed their current deworming scheme to be sufficient. Regarding the source of information on anthelmintic treatment, all dog owners indicated that they seek advice using the internet, whereas only 7 dog owners (1.4%) sought additional advice from veterinary staff.

Among the factors assessed for their association with deworming frequency in dogs, the frequency of vet visits and the owner’s attitude towards their pet had a statistically significant effect (Table 3). Dogs from owners who visited the vet more than once a year were treated approximately 1.39 times as often against helminths as dogs from owners who visited the vet once a year only (*P*-value < 0.01, Table 3). In addition, dogs from owners who had a sceptical attitude towards their dog were treated less often than those from affectionate owners (*P*-value: 0.036, Table 3). No significant differences between ESCCAP risk groups and no influence of neighbourhood and sources of information regarding the frequency of anthelmintic treatment in dogs were detected. Initially, the dog model showed a significant effect of the owner’s gender, with male owners deworming approximately 0.87 times less often per year than female owners [estimate: -0.143, standard error (SE): 0.068, *z*-value: -2.094, *P*-value: 0.036; model not shown]; however, this effect disappeared when the three data points with a deworming frequency of 12 times/year (outliers) were removed (Table 3).

**Table 3:** Results of the general linear model (GLM) with Poisson error structure and log link function testing the influence of various factors on annual deworming frequency in dogs

	Estimate	SE	95% CI	*z*-value	*P*-value
Intercept	0.719	0.307	0.087–1.296	2.343	**0.019**
Owner gender (ref: male, *n* = 182)	-0.129	0.070	-0.266–0.006	-1.855	0.064
Owner age	-0.001	0.002	-0.006–0.004	-0.492	0.623
Attitude towards pets					
Affectionate (*n* = 269)	Baseline				
Devoted (*n* = 126)	-0.100	0.077	-0.253–0.049	-1.305	0.192
Dispassionate (*n* = 53)	-0.074	0.114	-0.304–0.145	-0.649	0.516
Sceptical (*n* = 52)	-0.256	0.122	-0.502– -0.023	-2.101	**0.036**
Veterinary visits					
Once a year only (*n* = 227)	Baseline				
More than once a year (*n* = 273)	0.328	0.069	0.193–0.465	4.736	**<0.001**
German ESCCAP risk group^a^					
A (*n* = 10) *vs* B (*n* = 24)	-0.176	0.303	-0.932–0.581	-0.581	0.930
A (*n* = 10) *vs* C (*n* = 154)	0.008	0.261	-0.646–0.662	0.031	1.000
A (*n* = 10) *vs* D (*n* = 312)	-0.011	0.259	-0.657–0.636	-0.041	1.000
B (*n* = 24) *vs* C (*n* = 154)	0.184	0.178	-0.260–0.628	1.036	0.701
B (*n* = 24) *vs* D (*n* = 312)	0.165	0.172	-0.266–0.596	0.958	0.749
C (*n* = 154) *vs* D (*n* = 312)	-0.019	0.070	-0.194–0.156	-0.268	0.992
Neighbourhood^a^					
Rural (*n* = 186) *vs* city (*n* = 58)	0.003	0.106	-0.268–0.273	0.025	1.000
Suburban (*n* = 79) *vs* city (*n* = 58)	-0.051	0.124	-0.367–0.266	-0.411	0.976
Town (*n* = 177) *vs* city (*n* = 58)	-0.012	0.107	-0.286–0.261	-0.117	0.999
Suburban (*n* = 79) *vs* rural (*n* = 186)	-0.054	0.099	-0.306–0.199	-0.543	0.947
Town (*n* = 177) *vs* rural (*n* = 186)	-0.015	0.075	-0.208–0.177	-0.201	0.997
Town (*n* = 177) *vs* suburban (*n* = 79)	0.038	0.100	-0.218–0.295	0.383	0.980
Source of information regarding deworming					
Veterinarian/vet nurse (ref: yes, *n* = 7)	0.412	0.213	-0.029–0.811	1.928	0.054
Non-veterinarian (other pet owners, pet shop staff, etc.) (ref: yes, *n* = 331)	-0.093	0.073	-0.236–0.050	-1.278	0.201
Books and magazines (ref: yes, *n* = 93)	0.079	0.089	-0.098–0.252	0.889	0.374

*Note*: For this model, three outlier datapoints with a deworming frequency of 12 times/year were removed. The model was significantly different from a null model containing only an intercept term (likelihood ratio test, *χ*^2^ = 47.25, *df* = 15, *P* < 0.001). Significant *P*-values are printed in bold

^a^Multiple comparisons for the levels of ESCCAP risk group and neighbourhood using Tukey contrasts with single-step *P*-value adjustment were performed using the function glht from the package *multcomp* in R

*Abbreviations*: SE, standard error; CI, confidence interval; ref, reference

Regarding anthelmintic product use, 72.2% (361/500) of dog owners indicated that they had used at least one product effective against both tape- and roundworms during the past 12 months, while 16.2% (81/500) had only used a product effective against roundworms. Products that are only effective against tapeworms were not used by dog owners. The remaining 11.6% of dog owners indicated that they had used another product not contained in the list of currently licensed anthelmintic formulations.

### Cats

For cats, the most frequently reported risk factors according to their lifestyle were hunting (88%), catching prey (85%) and contact with children/elderly (67%, Table 2). Only 7 cats were under 6 months old.

According to the risk factor analysis, all cats were placed either in ESCCAP risk group A [indoor cat, 52.8% (264/500)] or risk group D [cat with unsupervised outdoor access, 47.2% (236/500)]. Significantly more cats in rural areas were placed in category D compared to cats in cities (Fisher’s exact test, odds ratio: 5.59, 95% confidence interval:  3.05–10.53, *P* < 0.001; Fig. 1b).

In this survey the average number of dewormings per year reported in cats was 1.72 ± 1.33 (mean ± SD:), which is significantly lower compared to dogs (Wilcoxon-Mann-Whitney U-test, *W* = 146750, *P* < 0.001). The distribution of deworming frequency in cats per ESCCAP risk group is depicted in Fig. 2b. In total, 83.8% (419/500) of cats were dewormed less often than recommended, while a small proportion of cats in risk group A [7.2% (19/264)] were treated more often than necessary based on ESCCAP guidelines. Only 2.6% (13/500) of cat owners provided anthelmintic treatment more than 4 times per year, while 16% (80/500) dewormed their cat 3–4 times/year. Eighty-five percent (425/500) of cat owners believed that their current deworming regime is sufficient. Regarding the source of information on anthelmintic treatment, all cat owners indicated that they seek advice using the internet, whereas only 6.4% (32/500) sought additional advice from veterinary staff.

In cats, as in dogs, a significant association between annual deworming frequency and vet visits as well as between deworming frequency and attitude towards the pet was detected (Table 4). The magnitude of the effect of vet visits was very similar compared to the dog dataset, with approximately 1.37 times as many dewormings in the group who visited the vet more than once a year compared to the group with one vet visit per year only (*P* < 0.001). Furthermore, in the cat dataset, a significant difference in deworming frequency according to ESCCAP risk group was found, with a 1.7 times higher deworming frequency in category D animals as opposed to category A animals (*P* < 0.001). In addition, significant differences according to neighbourhood were found, with more frequent dewormings in rural areas and towns as compared to cities (*P* = 0.002 and *P* = 0.034, respectively). Finally, owners who sought deworming advice from persons other than veterinary staff (e.g. other pet owners/pet shop staff/breeders) practiced significantly more frequent deworming than owners who did not seek this advice (*P* = 0.029).

**Table 4:** Results of the general linear model (GLM) with Poisson error structure and log link function testing the influence of various factors on annual deworming frequency in cats

	Estimate	SE	95% CI		*z*-value	*P*-value
Intercept	-0.133	0.162	-0.455–0.181		-0.817	0.414
Owner gender (ref: male, *n* = 311)	-0.031	0.073	-0.175–0.111		-0.425	0.671
Owner age	-0.002	0.003	-0.007–0.003		-0.766	0.444
Attitude towards pets						
Affectionate (*n* = 258)	Baseline					
Devoted (*n* = 113)	0.095	0.085	-0.073–0.260		1.117	0.264
Dispassionate (*n* = 77)	-0.226	0.106	-0.438– -0.022		-2.133	**0.033**
Sceptical (*n* = 52)	-0.006	0.119	-0.245–0.222		-0.047	0.963
Veterinary visits						
Once a year only (*n* = 345)	Baseline					
More than once a year (*n* = 155)	0.312	0.072	0.170–0.453		4.335	**<0.001**
German ESCCAP risk group						
A (*n* = 264)	Baseline					
D (*n* = 236)	0.515	0.074	0.371–0.661		6.982	**<0.001**
Neighbourhood^a^						
Rural (*n* = 154) *vs* city (*n* = 91)	0.410	0.117	0.110–0.710		3.491	**0.002**
Suburban (*n* = 90) *vs* city (*n* = 91)	0.305	0.129	-0.026–0.635		2.358	0.083
Town (*n* = 165) *vs* city (*n* = 91)	0.316	0.117	0.017–0.616		2.703	**0.034**
Suburban (*n* = 90) *vs* rural (*n* = 154)	-0.105	0.098	-0.356–0.146		-1.072	0.702
Town (*n* = 165) *vs* rural (*n* = 154)	-0.093	0.084	-0.310–0.123		-1.106	0.681
Town (*n* = 165) *vs* suburban (*n* = 90)	0.012	0.101	-0.246–0.269		0.114	0.999
Source of information regarding deworming						
Veterinarian/vet nurse (ref: yes, *n* = 32)	-0.056	0.170	-0.395–0.274		-0.327	0.743
Non-veterinarian (other pet owners, pet shop staff, etc.) (ref: yes, *n* = 329)	0.170	0.078	0.018–0.324		2.177	**0.029**
Books and magazines (ref: yes, *n* = 87)	-0.090	0.116	-0.323–0.132		-0.779	0.436

*Note*: The model was significantly different from a null model containing only an intercept term (likelihood ratio test, *χ*^2^ = 120.19, *df* = 13, *P* < 0.001). Significant *P*-values are printed in bold

^a^Multiple comparisons for the levels of neighbourhood using Tukey contrasts with single-step *P*-value adjustment were performed using the function glht from the package *multcomp* in R

*Abbreviations*: SE, standard error; CI, confidence interval; Ref, reference

Regarding anthelmintic product use, 62.2% (311/500) of cat owners indicated that they had used at least one product effective against tapeworms as well as roundworms during the past 12 months, while 19.8% (99/500) had only used a product effective against roundworms and 8.0% (40/500) only a product against tapeworms. The remaining 10.0% (50/500) of cat owners indicated that they used another product not contained in the list of currently licensed anthelmintic formulations.

## Discussion

In the present study, more than 93% of dogs were considered to belong to high-risk groups (30.8% category C, 62.4% category D) as according to the German adaptation of the ESCCAP guidelines, for which 12 anthelmintic treatments per year against tapeworms, and 4 or 12 treatments against roundworms, respectively, are recommended. In cats, non-supervised outdoor access placed almost half of the companion cats into the high-risk group category D. In this survey, the average deworming frequency was 2.07 times/year in dogs and 1.72 times/year in cats. Furthermore, in dogs no significant difference in deworming frequency was detected between risk groups. In cats, a significant difference between categories A and D was found; however, category D cats were only dewormed 1.7 times more often than cats in category A, i.e. only 2–3 times per year, as opposed to a recommended treatment frequency of 12 times per year. As a consequence, almost 98% of dogs and 84% of cats were dewormed less often than recommended based on ESCCAP guidelines. Thus, there is a clear mismatch between recommended and practiced frequency of anthelmintic treatment both in dogs and cats in our dataset, despite most pet owners believing that their current deworming scheme is sufficient. With the data collected in this survey concluding that high percentages of the dog and cat population are in the high-risk groups, it might be debated whether a recommended quarterly deworming is sufficient for those animals, for which no risk assessment can be performed. [3]

Low deworming frequencies have also been reported by studies from the Netherlands: A study on 916 household dogs was conducted on prevalence, risk factors and dog owners’ attitude towards deworming. According to the owners, 10.8% of dogs had never received any anthelmintic treatment, 21.5% were treated once a year, 19.3% twice a year, 11.6% three times a year, 16.2% four or more times a year and 12.8% were treated because of indication [3]. In a similar study, cat owners reported that 27.2% of cats had never received any anthelmintic treatment, 12.5% were treated once a year, 35.8% 2–3 times a year, and only 4.5% ≥ 4 times a year [6].

The reason for the low compliance regarding anthelmintic treatment may be that pet owners have insufficient knowledge on the zoonotic risks posed by canine and feline parasites and/or insufficient instruction on this topic by veterinarians. A recent survey among 206 German veterinary students revealed that only 68% considered the “one-health concept” as relevant for their later professional life [33], indicating that even future veterinarians may not be sufficiently aware of the zoonotic risk posed by companion animal parasites. In a survey conducted in Australia, very few veterinarians routinely discussed the zoonotic potential of pet parasites with clients [34]. It is thus not surprising that the majority of pet owners in the abovementioned studies indicated that they performed anthelmintic treatment for the sake of the pet’s health, rather than public health [3, 6]. However, because gastrointestinal helminths rarely cause clinical symptoms in adult pets, the owners of these animals may be less likely to recognize the risk to public health and to use anthelmintic treatment. A Portuguese study reported that 35% of 536 pet owners knew the meaning of the word zoonosis, but most of them were not aware of the possible transmission of parasites from their pets to themselves [4]. Similarly, while 49% of 185 Italian pet owners were aware of the risks for human health from canine and feline intestinal parasites, 36% believed that no risk exists and 15% declared that they had never considered such a possibility [7].

In addition to insufficient instruction by veterinarians regarding zoonoses and the implementation of effective control measures to reduce the risk of parasitic infections, pet owners might be reluctant to use anthelmintics because they want to avoid chemotherapeutic treatment options or possible adverse reactions. Regarding anthelmintic product use, the majority of pet owners (72.2% of dog and 62.2% of cat owners) in this survey indicated that they had used a product effective against both tape- and roundworms in the previous 12 months. However, approximately 10% of pet owners indicated that they used another product not contained in the list of currently licensed anthelmintic products for dogs and cats. Either these owners did not recognize the name of the product they had used, or they may have used herbal, homeopathic or other substances. Here, veterinarians also play a key role in educating pet owners about the safety of licensed anthelmintics and about effective and sustainable antiparasitic therapy and control strategies compared to the use of herbal or other “natural” products or homeopathic substances, for which evidence-based studies are missing. To achieve the goal of better implementation of expert recommendation, deeper insights into the barriers of pet owners to implement the recommended measures are necessary, as well as studies on the correct communication, following examples from the dairy industry [35].

Both in the dog and in the cat datasets, the number of yearly vet visits had a significant positive impact on deworming frequency. This is not self-explanatory, since in Germany not all veterinary medicinal products for deworming are obtained from a veterinarian and the survey did not discriminate between treatment at the vet and at home. However, only very few pet owners indicated that they actively seek advice on anthelmintic treatment from veterinarians and veterinary nurses. In the overall dataset, the mean annual deworming frequency in pets of owners who seek advice from their vet was 3.7 (dogs) and 1.8 (cats) as compared to 2.0 (dogs) and 1.7 (cats) of those owners who seek advice elsewhere. However, these differences were not statistically significant, probably since only seven dog owners and 32 cat owners reported that they seek veterinary advice on deworming. Since the survey participants were recruited *via* the internet, it makes sense that all respondents reported that they used the internet for seeking advice regarding deworming their pet.

Another reason for the low deworming frequency could be that many pets are only treated upon indication, i.e. after a positive coproscopic examination. Unfortunately, since in this survey no data were collected regarding whether or not deworming decisions were based on faecal examination results, this aspect cannot be assessed. However, in practice, the effort and costs for coproscopical analyses often exceed the effort and costs of deworming. Thus, faecal examination is only rarely performed and therefore this explanation is unlikely. Further investigations on the percentage of pet owners performing diagnosis *vs* pet owners deworming prophylactically are needed.

Furthermore, no correlation between ESCCAP risk group/deworming behaviour and actual parasitic burden can be made, since the parasitological status of the respondent’s pets was not assessed in the present study. Such data would certainly be highly worthwhile to also evaluate the agreement between the ESCCAP risk group assignment and actual infection status under the prevailing conditions in Germany. Nevertheless, previous studies have shown that a treatment frequency of less than four times per year is insufficient to reduce *Toxocara* spp. prevalence [36].

In a Portuguese study, cats and dogs were dewormed in a similar frequency [4]. However, our observation that cats seem to be dewormed less often than dogs is consistent with previous studies from the Netherlands [3, 6]. An explanation could be that cat owners may have a lower awareness regarding parasite infections than dog owners. A key reason for this could be that many more cats than dogs are kept without any outdoor access. Interestingly, cat owners performed more frequent anthelmintic treatments if they sought advice from other people (apart from veterinarians) compared to owners that did not seek this advice. This could be due to the fact that seeking advice from others probably raises their awareness, whereas dog owners already show a higher level of awareness regarding helminth infections and are thus less susceptible to advice from others. Of note, cats in rural areas and towns were dewormed more frequently compared to cats held in cities, independent of risk group assignment. This was not found for dogs. Possibly, there is a higher awareness regarding parasite infections in cat owners living in rural areas in Germany, as cats generally have more outdoor access in these areas. This was reflected by the fact that a significantly larger proportion of cats was assigned to the high-risk group D in rural areas than in cities. These results are in contrast to a Portuguese study about the awareness of pet owners regarding zoonoses, which detected no influence of the place of residence of the 536 responders to a questionnaire on having heard about and knowing the meaning of zoonosis [4].

A small proportion of cats in the risk group A [7.2%, (19/264)] were treated more often than necessary based on the factors considered here. However, for 16 of these 19 cats, contact with children was reported. Young children are especially at risk of acquiring zoonoses due to a less developed immune system and lower hygienic awareness than adults. Unfortunately, age of children was not assessed in the questionnaire. In case where young children were present, this factor would constitute a valid reason for the higher deworming frequency in indoor cats to prevent transmission of zoonotic parasites.

Further factors, which were not considered in our survey, might have an influence on deworming frequency. For example, owner level of education might have an effect. In a survey on Portuguese pet owners, the number of owners who were aware of the zoonotic potential of parasites was significantly higher in owners with intermediate and/or higher academic degree [4]. Nevertheless, in an Italian study, gender, age, education level of pet owners, family size and presence of children did not affect the occurrence of patent infections of the animals [7]. Furthermore, a previous diagnosis of a helminth infection in their pet might positively influence the owner’s deworming behaviour. To our knowledge, this aspect has not been considered in any study so far, and might be worthwhile exploring in the future.

Participants of the survey were not selected randomly, and therefore a possible bias in our dataset cannot be completely excluded. Participants that take part in a survey may be more interested in pet health topics than the basic population of pet owners. Because at least one vet visit per year was an inclusion criterion for this survey, it has to be expected that the deworming frequency in the general public is even lower than reported here.

## Conclusions

This survey reveals that based on their husbandry conditions and behaviour, many pets are at high risk of helminth infection. Notably, 62% of dogs and 47% of cats were assigned to category D for which monthly treatments/examinations are recommended by ESCCAP guidelines. Because of their zoonotic impact and their potential to cause clinical diseases in pets, education of pet owners regarding parasites through veterinarians and public health institutions is crucial for the reduction of risk exposure. The reported low deworming frequencies despite high potential parasite infection risk indicates that the knowledge of pet owners is insufficient to make sound decisions on routine deworming, and that instructing by veterinarians on this topic needs to be improved. Only a low percentage of pet owners actively sought information at veterinary practices, but the number of yearly veterinary visits had a significant positive impact on deworming frequency. Thus, this study highlights the importance of veterinary advice to pet owners about parasites and zoonoses. This advice should include an adequate risk assessment of each animal to derive a strategic deworming or faecal examination routine. Further studies should assess how communication between veterinarians and pet owners can be improved to increase owner compliance. Among veterinarians, awareness needs to be raised regarding the fact that it is their responsibility to protect not only the pet’s health from parasitic infections, but also that of the pet’s owners as well as the general public.

## Abbreviations

ESCCAP: European Scientific Counsel Companion Animal Parasites
GLM: generalized linear model
SD: standard deviation
SE: standard error
